# Preeclampsia prediction with maternal and paternal polygenic risk scores: the TMM BirThree Cohort Study

**DOI:** 10.1038/s41598-025-97291-x

**Published:** 2025-04-21

**Authors:** Hisashi Ohseto, Mami Ishikuro, Taku Obara, Akira Narita, Ippei Takahashi, Genki Shinoda, Aoi Noda, Keiko Murakami, Masatsugu Orui, Noriyuki Iwama, Masahiro Kikuya, Hirohito Metoki, Junichi Sugawara, Gen Tamiya, Shinichi Kuriyama

**Affiliations:** 1 Graduate School of Medicine, Tohoku University, Sendai, Miyagi Japan; 2 Tohoku Medical Megabank Organization, Tohoku University, Sendai, Miyagi Japan; 3 Tohoku University Hospital, Tohoku University, Sendai, Miyagi Japan; 4 Graduate School of Medicine, Teikyo University, Itabashi-ku, Tokyo, Japan; 5 Graduate School of Medicine, Tohoku Medical and Pharmaceutical University, Sendai, Miyagi Japan; 6 Suzuki Memorial Hospital, Iwanuma, Miyagi Japan; 7 RIKEN Center for Advanced Intelligence Project, Chuo-ku, Tokyo, Japan; 8 International Research Institute of Disaster Science, Tohoku University, Sendai, Miyagi Japan

**Keywords:** BirThree Cohort Study, Family history, Genome-wide association study, Hypertensive disorders of pregnancy, LDpred2, Medical genomics, Personalized medicine, Epidemiology, Genetics research

## Abstract

Genomic information from pregnant women and the paternal parent of their fetuses may provide effective biomarkers for preeclampsia (PE). This study investigated the association of parental polygenic risk scores (PRSs) for blood pressure (BP) and PE with PE onset and evaluated predictive performances of PRSs using clinical predictive variables. In the Tohoku Medical Megabank Project Birth and Three-Generation Cohort Study, 19,836 participants were genotyped using either Affymetrix Axiom Japonica Array v2 (further divided into two cohorts—the PRS training cohort and the internal-validation cohort—at a ratio of 1:2) or Japonica Array NEO (external-validation cohort). PRSs were calculated for systolic BP (SBP), diastolic BP (DBP), and PE and hyperparameters for PRS calculation were optimized in the training cohort. PE onset was associated with maternal SBP-, DBP-, and PE-PRSs and paternal SBP- and DBP-PRSs only in the external-validation cohort. Meta-analysis revealed overall associations with maternal PRSs but highlighted significant heterogeneity between cohorts. Maternal DBP-PRS calculated using “LDpred2” presented the most improvement in prediction models and provided additional predictive information on clinical predictive variables. Paternal DBP-PRS improved prediction models in the internal-validation cohort. In conclusion, Parental PRS, along with clinical predictive variables, is potentially useful for predicting PE.

**Supplementary Information:**

The online version contains supplementary material available at 10.1038/s41598-025-97291-x.

## Introduction

Preeclampsia (PE) is a multisystem disorder characterized by de novo hypertension and proteinuria, affecting approximately 3.4% and 2.7% of pregnant women in the USA^1^ and Japan^2^, respectively. It causes approximately 45,900 maternal deaths worldwide each year^3^. In addition to effective interventions, such as moderate exercise^4^, aspirin administration^5^, and calcium supplementation^6^, increased vigilance throughout the high-risk pregnancy can aid in the early detection and treatment of PE, potentially reducing adverse pregnancy outcomes and leading to better outcomes^7^ for mothers and fetuses. Therefore, developing accurate PE prediction models is crucial for clinical practice.

More than a hundred prediction models for PE are reported^8^, with some using genomic information from pregnant women (here, “maternal genomic information”)^9–12^. As the heritability of PE was estimated to be 55% in a family study, with maternal and fetal contributions accounting for 35% and 20%, respectively^13^, maternal genomic information is expected to provide a good biomarker for PE prediction. Integrating maternal genomic information as a polygenic risk score (PRS) can potentially improve existing predictive models; however, relevant research remains insufficient. In previous studies^9–12^, PRS for blood pressure (BP) and PE were utilized but variables such as actual measured BP values and family history of PE, which are important clinical predictive variables, were not simultaneously incorporated into the prediction models. Actual measured BP values in early pregnancy are an effective PE biomarker and are included in many clinical prediction models^8,14^. Similarly, family medical history is a predictive variable for diseases and reflects the genetic load and shared environmental factors^15,16^. A recent study revealed that the PRS and family medical history provide complementary information on noncommunicable diseases^17^. As actual measured BP values and family history of hypertensive disorders of pregnancy (HDP) are more accessible than PRS, their combined clinical usefulness with genomic information should be explored. Although a previous study^18^ reported no improvement when PRS was incorporated into a machine learning-based prediction model utilizing clinical predictive variables, this study had a small number of cases (< 100) and was not externally validated.

Given that genomic information from the paternal parent of the fetus (here, “paternal genomic information”) is transmitted to both fetal and placental tissues, the paternal genomic information holds predictive potential for PE. As obtaining the fetal genome is difficult, combining the maternal and paternal genomes may capture a comprehensive genetic predisposition to PE. However, no previous studies have employed such information in PE prediction models.

Herein, we examined the relationship of maternal and parental genomic information with PE onset. Additionally, we investigated whether parental PRSs have predictive information in addition to clinical models, including actual measured BP values and family history of HDP.

## Methods

### Participants

The Tohoku Medical Megabank (TMM) Project Birth and Three-Generation Cohort Study (the BirThree Cohort Study)^19,20^ recruited pregnant women and their families between 2013 and 2017. More than 50 obstetric clinics and hospitals in the Miyagi Prefecture, Japan, participated, registering 23,406 pregnant women and 8,823 participants who self-reported as fathers of the fetuses.

We excluded the following pregnant women from the study: those who withdrew consent, had multiple pregnancies, had stillbirth before 20 weeks of gestation, and missed the diagnosis of PE or delivery date. Herein, we included only the first-time participations of those who had multiple participations in the TMM BirThree Cohort Study.

**Fig. 1:**
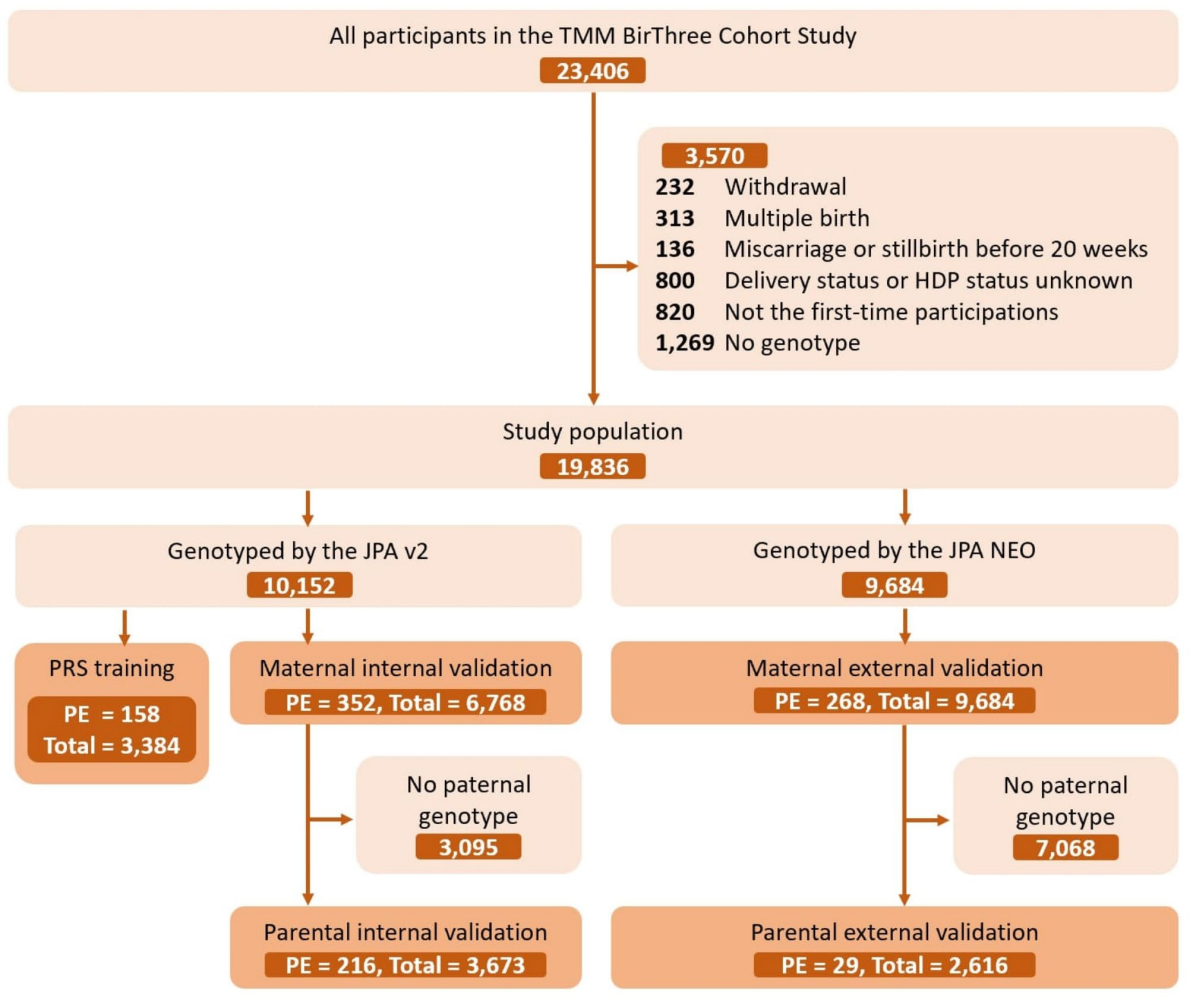
TMM BirThree Cohort Study flow for three study cohorts. After excluding ineligible participants, the cohort was divided into three cohorts, namely PRS training, maternal internal validation, and maternal external validation. The cohorts with paternal genotypes in the maternal internal and external validation cohorts were the parental internal and external validation cohorts, respectively. The numbers of PE cases and total participants in each cohort are indicated in the format “PE cases, total participants”. TMM BirThree Cohort Study: The Tohoku Medical Megabank Project Birth and Three-Generation Cohort Study; JPA v2: Affymetrix Axiom Japonica Array v2; JPA NEO: Affymetrix Axiom Japonica Array NEO; PRS, polygenic risk score; and HDP, hypertensive disorders of pregnancy.

Participants were genotyped using either the Affymetrix Axiom Japonica Array v2 (JPA v2) or Japonica Array NEO (JPA NEO), depending on the timing of their genotyping within the TMM Project^21^. Participants analyzed earlier in the genotyping process were assigned to JPA v2, while those analyzed later were assigned to JPA NEO. Therefore, differences in the proportions of recruitment regions, locations, and the rate of missing family data may exist between the two genotyping platforms. Those with missing genotyping data were excluded after a standard quality control procedure^22^. Those genotyped using JPA v2 were divided into two cohorts—the PRS training cohort and the maternal internal validation cohort—at a ratio of 1:2. Those genotyped using JPA NEO were defined as the maternal external validation cohort. The paternal parents of the fetuses were genotyped using the same genotyping array platform as the participants. Cohorts with paternal genotypes in the maternal internal and external validation cohorts were defined as the parental internal and external validation cohorts, respectively (Fig. 1). Ethical approval was obtained from the Ethics Committee of the Tohoku Medical Megabank Organization (2013-1-103-1 and 2023-4-025), and informed consent for research participation was obtained from all participants. This study was performed in accordance with the Declaration of Helsinki.

### Predictive variables

Based on previous research on PE prediction models^23–27^, we selected the following predictive variables: maternal age at conception, pre-pregnancy body mass index (BMI), chronic hypertension (CH), systemic lupus erythematosus (SLE), type 1 and 2 diabetes mellitus (DM), maternal family (mother or sisters) history of HDP, conception via in vitro fertilization (IVF), parity (nulliparous, parous with or without previous HDP), gestational age at the previous delivery, the inter-birth interval, and BP at the first antenatal care during 10–13 weeks of gestation. We collected BP data at 10–13 weeks of gestation when the majority of the population underwent the first or second antenatal care. Mean arterial pressure (MAP) was calculated and converted to log_10_ transformed multiple of the median (log MoM) for the prediction model. The median was chosen as the scaling factor due to the positively skewed data distribution, aligning with prior research for consistency and comparability^23–27^. Paternal age and family history of HDP were obtained for parental analyses.

### Polygenic risk score

Genotyping and PRS calculations are described in Supplementary Methods. In brief, PRSs for three phenotypes, systolic BP (SBP), diastolic BP (DBP), and PE, were calculated using two methods, genome-wide clumping and thresholding (C + T) and a Bayesian approach using LDpred2^28,29^. Although LDpred2 is generally considered more accurate than C + T, C + T is easier to implement and interpret.^27^ The hyperparameters for maternal PRS were optimized in the PRS training cohort and the same parameters were applied to the paternal PRS for optimization. The number of variants included in each PRS is summarized in Supplementary Table S1.

### Outcome measurement

PE was identified according to the guidelines of the American College of Obstetricians and Gynecologists^30,31^, using medical records at antenatal care. PE superimposed on the CH was included in the definition of PE. PE was automatically diagnosed using an algorithm based on medical record data and validated by a physician. Additionally, PE was classified as early or late onset based on if it occurred earlier than 34 weeks of gestation. The details are given elsewhere^24,30^.

### Model development

Herein, the competing risk model^32^ was applied, which has been validated both internally and externally in Europe^33^ and yielded comparable results in the TMM BirThree Cohort Study^23,24^. This model assumes that all pregnant women are at risk of developing PE during pregnancy. However, some women can give birth before PE can develop. This case, where delivery occurs before the onset of PE, is considered a competing event. Such competing events can influence the prediction model’s accuracy by reducing the number of observed PE cases. A competing risk model is particularly suited for this scenario because it accounts for these competing events, providing a more accurate estimation of the probability of PE onset compared to traditional models that assume no such competition. A parametric survival model with a Gaussian distribution was applied^32^ and delivery without PE was considered censored. Missing predictive variables were imputed using k-nearest neighbor imputation with k = 140 (square root of the total study population^34^). To assess the model discrimination, Harrell’s C-statistic was calculated for the whole gestational age. To assess model calibration, the calibration slope was calculated by regressing observed survival outcomes on predicted gestational age at delivery with PE in the same parametric survival models^35^.

### Statistical analysis

Herein, baseline characteristics were compared by PE status or internal/external validation cohorts. *P*-values were calculated using the *t*-test for continuous variables and the chi-square test or Fisher’s exact test for categorical variables. Pearson’s correlation coefficients were calculated for all combinations of parental PRSs using LDpred2 in the parental internal validation cohort.

Association analyses were performed using SBP-PRS, DBP-PRS, and PE-PRS using LDpred2. In the maternal internal and external validation cohorts, the associations between maternal PRSs and PE onset were examined using logistic regression analysis adjusted for maternal age and four genetic principal components (PC), with parameters estimated using the Maximum Likelihood Estimation method. Two models were developed, one with PRSs as continuous values and the other as tertile values. In the parental internal and external validation cohorts, associations between both maternal and paternal PRSs and PE onset were examined using logistic regression analysis adjusted for parental age and four genetic PCs. Maternal and paternal PRSs for the same phenotype were simultaneously included in the model. The results of internal and external validation cohorts were merged using inverse-variance weighting for meta-analysis. Interactions between maternal and paternal PRSs were also examined. As subanalyses, participants with CH were excluded from the study population, and analyses were performed again. To further assess the robustness of the results, linear regression analyses were performed with PRSs as exposures and BP in early pregnancy as the outcome. Additionally, the early- and late-onset PE were analyzed as outcomes to determine if the association with PRS differed by PE pathology.

The prediction model was developed in two stages: identification of a suitable PRS for prediction models and development of clinical prediction models using parental PRSs. In the first stage, prediction models were developed using each maternal PRS, age, and four genetic PCs as continuous variables. Based on the C-statistics of prediction models, we selected either C + T or LDpred2 as the PRS calculation algorithm, and SBP-PRS, DBP-PRS, or PE-PRS as the PRS phenotype. Four prediction models were developed in the second stage: models with maternal age (the reference model), models with maternal age and maternal family history of HDP (the family-history model), models with variables available at pregnancy confirmation such as maternal age, pre-pregnancy BMI, CH, SLE, DM, maternal family history of HDP, conception via IVF, parous with or without previous PE, gestational age at the previous delivery, and the inter-birth interval (the at-pregnancy-confirmation model), models with variables available in early pregnancy such as variables included in the at-pregnancy-confirmation model and BP in early pregnancy (the in-early-pregnancy model). C-statistics and calibration slopes were calculated for each model. Model training and internal validation were conducted using the internal validation cohort. Apparent performance, which reflects the raw performance of the model trained on the internal validation cohort and may be overestimated, was also reported. External validation was conducted using the external validation cohort. The bootstrap method with 5,000 resamplings was used for two purposes: (1) to perform internal validation of the prediction models and (2) to calculate the 95% confidence intervals (CIs) for apparent and external validation results. This dual application ensures robustness in internal validation and accurate estimation of CIs across validation cohorts. *P*-values represent comparisons between models with and without PRSs. A simulation study was performed to confirm the reproducibility of the first step. The cohort genotyped with JPA v2 was divided into PRS training and internal validation cohorts, with split ratios of 1:2, 2:3, and 1:1, repeated with 100 random numbers, to determine the PRS with the best predictive ability.

All analyses were conducted using R (4.1.0) unless otherwise noted. We considered a two-tailed *P*-value of < 0.05 as significant.

## Results

A total of 19,836 participants were eligible for the present study: 3,384 in the PRS training cohort, 6,768 in the maternal internal validation cohort, and 9,684 in the maternal external validation cohort (Fig. 1). In the maternal internal and external validation cohorts, 3,673 (54.3%) and 2,616 (27.0%) participants, respectively, had paternal genotyping data.

In total, 352 of 6,768 (5.2%) and 268 of 9,684 (2.8%) participants developed PE in the maternal internal and external validation cohorts, respectively (Table 1). Of the 3,673 and 2,616 participants in the parental internal and external validation cohorts, respectively, 216 (5.9%) and 29 (1.1%) participants developed PE (Supplementary Table S2). Participants with PE tended to have an earlier delivery; were older; had a higher BMI; had a history of CH, DM, and SLE; were nulliparous or parous with previous PE; had a shorter last delivery gestational age; conceived via IVF; and had a higher BP at 10–13 weeks of gestation, with slight variations among the maternal and parental cohorts (Table 1 and Supplementary Table S2). Notable differences in the characteristics between the internal and external validation cohorts were observed (Supplementary Tables S3 and S4). Strong correlations existed between maternal PRSs or among paternal PRSs, whereas weak correlations existed between maternal and paternal PRSs (Supplementary Figure S1).

In association studies on the maternal cohorts (Table 2), maternal SBP-, DBP-, and PE-PRSs were significantly associated with PE onset in the meta-analysis; odds ratio (OR) and 95% CI per 1 standard deviation (SD): 1.14 (1.05 to 1.24), 1.20 (1.11 to 1.31), and 1.10 (1.01 to 1.19), respectively. These significant associations were primarily driven by the external validation cohort, as the internal validation cohort showed weaker and non-significant associations. Significant heterogeneity was observed (P for heterogeneity = 0.001, 0.003, and 0.030, respectively). In association studies for the parental cohorts (Supplementary Table S5), maternal and paternal PRSs were not related to PE onset in the meta-analysis. Only in the parental external validation cohort, paternal SBP-PRS and paternal DBP-PRS were associated with PE onset; OR and 95% CI per 1 SD: 1.93 (1.32 to 2.83) and 1.91 (1.29 to 2.84), respectively. Similar results were observed after excluding participants with CH. However, some results did not converge in the parental external validation cohort because of the small number of outcomes (Supplementary Tables S6 and S7). No interaction was noted between the maternal and paternal PRSs (data not shown). The associations of PRSs with BP during early pregnancy were similar to their associations with PE (Supplementary Table S8). When early- and late-onset PE were compared in relation to PRS, the effect sizes were generally comparable (Supplementary Table S9).

After parameter optimization in the PRS training cohort, the predictive performances of SBP-, DBP-, and PE-PRS calculated using the two methods, C + T and LDpred2, were compared in the maternal internal and external validation cohorts (Table 3). The DBP-PRS calculated using LDpred2 improved the discrimination performance the most in both the internal (0.570 in the model with PRS versus 0.567 in the model without PRS, *P*-value = 0.200) and external (0.592 in the model with PRS versus 0.551 in the model without PRS, *P*-value < 0.001) validation. The simulation study revealed that among the PRSs predicted by LDpred2, those for DBP were the most frequent with the highest predictive power in the external validation cohort (Supplementary Table S10).

Thereafter, clinical prediction models were developed with maternal and paternal DBP-PRSs calculated using LDpred2. In the maternal cohort, models including maternal PRS demonstrated potential superior predictive discrimination compared to models without maternal PRS in the reference and family-history models in internal validation, although these differences were not statistically significant. This was also observed in all four external validation models, namely the reference, family-history, at-pregnancy-confirmation, and in-early-pregnancy models (Table 4). However, the differences in predictive performance between models with and without maternal PRS were not statistically significant in all cases, indicating limited evidence of robust improvement in discrimination ability. For example, in external validation, the C-statistics of the at-pregnancy-confirmation model were 0.777 without PRS and 0.784 with maternal PRS (*P*-value = 0.242), indicating the possible utility of maternal PRS at pregnancy confirmation. Even in the in-early-pregnancy model, which included BP in early pregnancy, the C-statistics improved slightly by including maternal PRS (0.849 without PRS versus 0.851 with maternal PRS, *P*-value = 0.520), suggesting the possible utility of maternal PRS in the prediction of early pregnancy. In the parental internal validation cohort (Table 5), paternal PRS, but not maternal PRS, improved the reference, family-history, and at-pregnancy-confirmation models (*P*-value = 0.018, 0.059, and 0.022, respectively). In the parental external validation cohort, maternal, but not paternal, PRS improved the reference, family-history, and at-pregnancy-confirmation models (*P*-value = 0.568, 0.549, and 0.410, respectively), although C-statistics had a wide 95% CI and the results were not stable or statistically significant. Models with both maternal and paternal PRSs did not demonstrate better discrimination ability than those with either maternal or paternal PRS (Table 5).

## Discussion

The maternal SBP-, DBP-, and PE-PRSs were associated with PE onset in the meta-analysis, with significant associations observed only in the external validation cohort, reflecting heterogeneity across cohorts. The paternal SBP- and DBP-PRSs were associated with PE onset only in the external validation cohort. Maternal DBP-PRS calculated using LDpred2 improved prediction models the most. Maternal DBP-PRS improved both at-pregnancy-confirmation and in-early-pregnancy models, indicating the clinical utility of maternal PRS. Paternal PRS improved prediction models in the parental internal validation cohort. These results suggest the potential for PRSs to improve predictive performance, but the lack of statistical significance in some cases highlights the need for cautious interpretation and further validation in larger, diverse cohorts.

### Maternal PRS

The present study shows that maternal PRSs for BP were associated with PE onset in the external validation cohorts, consistent with previous studies in the European population^9,10^, indicating cross-ethnicity generalizability. However, the internal-validation cohort showed weaker and non-significant associations. This discrepancy may be partly explained by differences in baseline characteristics (Supplementary Table S3) and genotyping platform, as discussed later. Regarding the onset time, the association between PRS and PE was not considerably affected, suggesting that PRSs are not pathology-specific biomarkers and broadly capture the genetic predisposition to hypertension. Maternal PRS improved the prediction model beyond the family history of HDP. While family history provides plentiful information on genetic predisposition and environment^15^, the amount of information is determined by family structure and relationships, as well as by disease prevalence. Owing to the demographic trend toward smaller families^36^ and the need to obtain data retrospectively because of the transient nature of the condition during pregnancy, obtaining a comprehensive family history of HDP is difficult. Herein, < 2% of the participants and < 1% of the paternal parents of their fetuses reported a family history of HDP. Moreover, reliance only on the binary categorization of family history may result in overlooking the intricate genetic predisposition. Therefore, incorporating PRSs into risk prediction models in addition to family history is crucial for precision medicine. Maternal PRS improved the at-pregnancy-confirmation model and slightly improved the in-early-pregnancy model. This indicates that the maternal PRS is useful in clinical practice at pregnancy confirmation and even in early pregnancy, although its predictive significance is diminished by the fact that some genetic predisposition for high BP may manifest itself as the actual measured BP values in early pregnancy. In contrast, PE-PRS was significantly associated with the development of PE in the meta-analysis, though the association was relatively modest compared to SBP- and DBP-PRSs. The genome-wide association study (GWAS) for PE^37^ used in our study had a relatively small number of cases, and the number of East Asian populations in the GWAS was limited (123 in the BioBank Japan, 1,031 in the UK Biobank, and 1,324 in the FinnGen), which might result in failure to reflect genetic PE predisposition.

### Paternal PRS

Furthermore, in this study, paternal PRSs for BP were associated with PE onset in the external validation cohort. The paternal genetic contribution to PE onset has been predicted in family^13^ and genetic studies^38^. However, as of date, the association between paternal PRS and PE onset has not been investigated. Our results were inconsistent between the internal and external validation cohorts. One possible reason for this is the biological differences between the parental internal and external validation cohorts. Placental and maternal factors are involved in the development of PE^39^. The baseline characteristics of the parental internal and external cohorts were quite different; the prevalence of CH in participants with PE was much higher in the external validation cohort than in the internal validation cohort (79.3% versus 25.0%). This may have led to differences between the two cohorts in terms of the ratio of placental to maternal contributions, resulting in different associations between paternal PRSs and PE. In addition to these biological differences, differences in genotyping platforms between the internal (JPA v2) and external (JPA NEO) validation cohorts may also have contributed to the observed discrepancies^21^. Both JPA v2 and JPA NEO arrays share common SNPs after imputation, ensuring consistency in PRS calculations. However, the genotyping and imputation quality metrics differ due to advances in the design of JPA NEO. While JPA v2 used mutual information for tag SNP selection, JPA NEO adopted a more standardized protocol using pairwise r² for selecting tag SNPs^21^. JPA NEO showed a greater number of variants with INFO scores > 0.8 compared to JPA v2 highlighting improved imputation accuracy driven by advancements in its design and reference panel^21^. These advancements in JPA NEO likely contributed to the observed differences in predictive performance between the cohorts. Paternal PRS improved the prediction models in the parental internal validation cohort, despite no significant association in the association analyses. The prediction models developed in the parental internal validation cohort did not have predictive power in the parental external validation cohort possibly, because of cohort differences, as mentioned previously. This suggests that the contribution of paternal PRS to PE onset may differ across populations and should be considered when developing predictive models. We also observed significant decreases in the C-statistics when including paternal PRS in the external validation cohort. One possible reason is that adding a variable with limited predictive value can sometimes introduce noise rather than improving accuracy, ultimately destabilizing the model. Another possibility is that, in the external validation cohort, the association observed in the internal validation cohort may not have held, causing paternal PRS to introduce misleading information and reduce predictive performance. Furthermore, the near-perfect prediction observed in the at-pregnancy-confirmation and in-early-pregnancy models in the parental external validation cohort is likely driven by the high prevalence of CH. CH is the strongest risk factor for PE, and its overrepresentation simplifies prediction. Additionally, the small number of PE cases in the parental external validation cohort (29 cases) may lead to unstable estimate.

### Clinical and research implications

Risk prediction is crucial in clinical practice because it can lead to preventive interventions, allowing for intensive perinatal management, which facilitates early detection and treatment. Genomic information remains unchanged and can be used throughout life. This is a crucial difference from other biomarkers^26^, which are only reflective of temporary conditions. In addition, PRS is useful for predicting diseases other than those in the perinatal period^40,41^. Given the unchangeability of genomic information, our study suggests that genomic information should be obtained during relatively healthy childbearing age rather than during middle or old age to avoid poor outcomes in pregnant women due to inadequate prediction of PE. We showed that maternal PRS can improve clinical PE prediction models and suggest its application in other perinatal diseases. In addition, we showed that the utility of paternal genomic information in predicting PE is inconsistent across cohorts; therefore, further studies should aim at identifying populations in which paternal PRS has predictive information for PE.

### Strengths and limitations

Our study is the first to investigate the utility of maternal and paternal PRS for PE prediction. We applied a well-validated prediction model, and while the observed improvements in predictive performance were modest, our findings provide a preliminary step toward clinical application. Two cohorts with genomic data from different genotype array platforms and baseline characteristics enabled us to examine the generalizability of the results.

Despite these strengths, our study had four limitations. First, our model did not include effective biomarkers specific to PE, such as the uterine artery pulsatility index and placental growth factor^26^, which may have improved the performance. However, given the widespread use of genomic information beyond perinatal diseases, prediction models using PRS may be more clinically applicable than those using disease-specific biomarkers. Second, there were several differences between the internal and external validation cohorts. The external validation cohort included more participants with CH, which was the strongest predictive variable, than the internal validation cohort, resulting in calibration slopes of > 1 in the external validation. Moreover, in the external cohort, a family history of HDP was not associated with PE, limiting the purpose of this study to confirm the predictive value of the PRS in addition to a family history of HDP. The differences between the two cohorts occurred possibly because of differences in the source populations, and although the study does not provide sufficient replication, it does provide some insight into the type of population for which the predictive model is useful. Third, because there is no paternal GWAS on PE, we had no choice but to apply the maternal GWAS to the fathers. Fourth, owing to the small number of parental cohorts, the effectiveness of paternal PRS in predicting PE may have been overlooked. Optimization was conducted only for maternal PRS, which may have resulted in an underestimation of the ability of paternal PRS to predict PE.

## Conclusions

Parental PRS, along with clinical predictive variables, is potentially useful for predicting PE.

**Table 1:** Baseline characteristics in the maternal internal and external cohort, stratified by the preeclampsia status.

	Maternal internal validation cohort (*n* = 6,768)				Maternal external validation cohort (*n* = 9,684)			
	PE	Not affected	*P*-value	*n* of missing	PE	Not affected	*P*-value	*n* of missing
	*n* = 352	*n* = 6,416			*n* = 268	*n* = 9,416		
Gestational age, weeks	38.6 ± 2.2	39.2 ± 1.6	< 0.001	0	38.3 ± 2.7	39.1 ± 1.8	< 0.001	0
Maternal age at conception, years	31.9 ± 5.6	31.5 ± 5.0	0.143	0	32.2 ± 5.2	31.4 ± 5.0	0.005	0
Pre-pregnancy BMI, kg/m^2^	23.6 ± 4.9	21.6 ± 3.4	< 0.001	113	23.5 ± 5.1	21.4 ± 3.3	< 0.001	124
CH, %	104 (29.5)	177 (2.8)	< 0.001	0	103 (38.4)	192 (2.0)	< 0.001	0
DM (type 1 or 2), %	8 (2.3)	17 (0.3)	< 0.001	2,162	0 (0.0)	9 (0.1)	> 0.999	3,818
SLE, %	2 (0.6)	5 (0.1)	0.048	2,162	2 (0.7)	3 (0.0)	0.007	3,818
Maternal family history of HDP, %	12 (3.4)	136 (2.1)	0.155	2,162	3 (1.1)	158 (1.7)	0.632	3,818
Parity			< 0.001	30			< 0.001	167
Nulliparous, %	176 (50.0)	2,796 (43.6)			102 (38.1)	3,615 (38.4)		
Parous with previous PE, %	30 (8.5)	174 (2.7)			29 (10.8)	260 (2.8)		
Parous with no previous PE, %	146 (41.5)	3,446 (53.7)			137 (51.1)	5,541 (58.8)		
Inter-birth interval, years	3.9 ± 3.5	3.6 ± 2.6	0.166	206	3.8 ± 2.9	3.6 ± 2.6	0.304	399
Last delivery gestational age, weeks	38.6 ± 2.1	39.0 ± 1.6	< 0.001	1,257	38.4 ± 1.8	39.0 ± 1.6	< 0.001	2,020
Conception by IVF, %	33 (9.4)	298 (4.6)	< 0.001	62	19 (7.1)	415 (4.4)	0.052	335
MAP at 10–13 weeks of gestation, mmHg	89.3 ± 11.5	80.4 ± 9.3	< 0.001	973	91.8 ± 11.7	79.6 ± 9.3	< 0.001	1,328

PE: preeclampsia; BMI: body mass index; CH: chronic hypertension; DM: diabetes mellitus; SLE: systemic lupus erythematosus; HDP: hypertensive disorders of pregnancy; IVF: in vitro fertilization; and MAP: mean arterial pressure. Data are shown as mean ± standard deviation for continuous variables and n (%) for categorical variables. *P*-values were calculated using the chi-square or Fisher’s exact test for categorical variables and the t-test for continuous variables. Missing values were imputed using the k-nearest neighbor imputation with k = 140 (square root of the total study population).

**Table 2:** Relationship between maternal polygenic risk scores and preeclampsia onset in the maternal cohorts by logistic regression analysis.

	Internal validation cohort		External validation cohort		Meta-analysis		
	OR (95% CI)	*P*-value	OR (95% CI)	*P*-value	OR (95% CI)	*P*-value	P for heterogeneity
PRS for SBP							
Continuous	1.01 (0.91 to 1.13)	0.794	1.33 (1.17 to 1.50)	< 0.001	1.14 (1.05 to 1.24)	0.002	0.001
Tertile 1	Reference		Reference		Reference		
Tertile 2	1.09 (0.83 to 1.44)	0.532	1.02 (0.76 to 1.39)	0.874	1.06 (0.86 to 1.30)	0.570	0.761
Tertile 3	0.96 (0.73 to 1.26)	0.768	1.61 (1.21 to 2.16)	0.001	1.22 (1.00 to 1.49)	0.046	0.010
PRS for DBP							
Continuous	1.08 (0.96 to 1.21)	0.186	1.39 (1.22 to 1.58)	< 0.001	1.20 (1.11 to 1.31)	< 0.001	0.003
Tertile 1	Reference		Reference		Reference		
Tertile 2	0.80 (0.59 to 1.08)	0.150	1.33 (0.98 to 1.79)	0.063	1.03 (0.84 to 1.28)	0.768	0.020
Tertile 3	1.05 (0.80 to 1.37)	0.748	2.18 (1.62 to 2.94)	< 0.001	1.46 (1.19 to 1.78)	< 0.001	0.000
PRS for PE							
Continuous	1.01 (0.91 to 1.13)	0.817	1.21 (1.07 to 1.37)	0.002	1.10 (1.01 to 1.19)	0.026	0.030
Tertile 1	Reference		Reference		Reference		
Tertile 2	1.05 (0.81 to 1.35)	0.705	1.34 (0.96 to 1.88)	0.085	1.15 (0.94 to 1.41)	0.179	0.252
Tertile 3	1.04 (0.79 to 1.36)	0.792	1.49 (1.08 to 2.05)	0.014	1.20 (0.98 to 1.48)	0.076	0.087

PRS: polygenic risk score; PE: preeclampsia; OR: odds ratio; CI: confidence interval; SBP: systolic blood pressure; and DBP: diastolic blood pressure. Two models were developed, one with PRS as a continuous value and the other as tertile values. All results were adjusted for maternal age at conception and four genetic principal components. P for heterogeneity was calculated by the Cochran Q test.

**Table 3:** Prediction performance with maternal polygenic risk score derived from three phenotypes using two methods.

	SBP-PRS		DBP-PRS		PE-PRS		No PRS
	LDpred2	C + T	LDpred2	C + T	LDpred2	C + T	
C-statistics							
Apparent	0.573 (0.539 to 0.607), *P*-value = 0.975	0.573 (0.540 to 0.607), *P*-value = 0.846	0.579 (0.545 to 0.613), *P*-value = 0.441	0.574 (0.541 to 0.608), *P*-value = 0.534	0.576 (0.543 to 0.610), *P*-value = 0.381	0.573 (0.540 to 0.607), *P*-value = 0.748	0.573 (0.539 to 0.607)
Internal validation	0.566 (0.550 to 0.574), *P*-value = 0.82	0.566 (0.548 to 0.577), *P*-value = 0.877	0.570 (0.554 to 0.579), *P*-value = 0.200	0.566 (0.549 to 0.577), *P*-value = 0.823	0.566 (0.549 to 0.577), *P*-value = 0.838	0.565 (0.547 to 0.574), *P*-value = 0.514	0.567 (0.550 to 0.575)
External validation	0.565 (0.521 to 0.606), *P*-value < 0.001	0.547 (0.504 to 0.589), *P*-value = 0.209	0.592 (0.551 to 0.632), *P*-value < 0.001	0.545 (0.501 to 0.586), *P*-value = 0.028	0.558 (0.514 to 0.600), *P*-value = 0.081	0.552 (0.508 to 0.593), *P*-value = 0.601	0.551 (0.507 to 0.593)
Calibration slope							
Apparent	0.997 (0.568 to 1.386)	0.996 (0.575 to 1.394)	1.000 (0.597 to 1.371)	0.997 (0.570 to 1.392)	0.998 (0.572 to 1.394)	0.997 (0.571 to 1.394)	0.996 (0.570 to 1.396)
Internal validation	0.828 (0.606 to 1.270)	0.826 (0.605 to 1.270)	0.834 (0.619 to 1.246)	0.824 (0.609 to 1.256)	0.824 (0.603 to 1.263)	0.825 (0.606 to 1.259)	0.859 (0.628 to 1.347)
External validation	0.960 (0.462 to 1.408)	0.806 (0.291 to 1.272)	1.116 (0.645 to 1.541)	0.786 (0.273 to 1.254)	0.903 (0.393 to 1.367)	0.848 (0.342 to 1.312)	0.846 (0.338 to 1.311)

PRS: polygenic risk score. PRSs were calculated for three phenotypes, systolic blood pressure (SBP), diastolic blood pressure (DBP), and preeclampsia (PE), using two methods, LDpred2 and C + T. Models without PRS included maternal age and four genetic principal components as predictive variables, and the others included additional PRSs. Model training and internal validation were conducted using the internal validation cohort. Apparent performance, which reflects the raw performance of the model trained on the internal validation cohort and may be overestimated, was also reported. External validation was conducted using the external validation cohort. *P*-values and 95% confidence intervals were obtained by the bootstrap method with 5,000 resamplings.

**Table 4:** Performance of prediction models with maternal polygenic risk score, baseline characteristics, and blood pressure in early pregnancy in the maternal cohorts.

	No PRS	M-PRS
C-statistics		
Apparent		
Reference model	0.561 (0.531 to 0.591)	0.579 (0.545 to 0.613), *P*-value = 0.171
Family-history model	0.564 (0.534 to 0.594)	0.579 (0.545 to 0.613), *P*-value = 0.224
At-pregnancy-confirmation model	0.736 (0.702 to 0.769)	0.742 (0.709 to 0.773), *P*-value = 0.301
In-early-pregnancy model	0.782 (0.751 to 0.810)	0.783 (0.753 to 0.812), *P*-value = 0.514
Internal validation		
Reference model	0.561 (0.561 to 0.561)	0.570 (0.554 to 0.579), *P*-value = 0.214
Family-history model	0.564 (0.559 to 0.565)	0.571 (0.556 to 0.580), *P*-value = 0.252
At-pregnancy-confirmation model	0.730 (0.715 to 0.737)	0.729 (0.713 to 0.739), *P*-value = 0.986
In-early-pregnancy model	0.777 (0.765 to 0.782)	0.776 (0.764 to 0.782), *P*-value = 0.86
External validation		
Reference model	0.577 (0.543 to 0.612)	0.592 (0.551 to 0.632), *P*-value = 0.317
Family-history model	0.572 (0.538 to 0.607)	0.585 (0.544 to 0.625), *P*-value = 0.352
At-pregnancy-confirmation model	0.777 (0.741 to 0.811)	0.784 (0.750 to 0.818), *P*-value = 0.242
In-early-pregnancy model	0.849 (0.820 to 0.875)	0.851 (0.821 to 0.876), *P*-value = 0.520
Calibration slope		
Apparent		
Reference model	0.998 (0.529 to 1.424)	1.000 (0.597 to 1.371)
Family-history model	0.996 (0.551 to 1.406)	0.997 (0.610 to 1.358)
At-pregnancy-confirmation model	0.999 (0.894 to 1.111)	0.999 (0.895 to 1.109)
In-early-pregnancy model	0.999 (0.903 to 1.101)	0.999 (0.903 to 1.101)
Internal validation		
Reference model	1.002 (0.701 to 1.883)	0.834 (0.619 to 1.246)
Family-history model	0.964 (0.694 to 1.684)	0.835 (0.631 to 1.212)
At-pregnancy-confirmation model	0.953 (0.717 to 1.071)	0.942 (0.723 to 1.056)
In-early-pregnancy model	0.956 (0.793 to 1.067)	0.946 (0.784 to 1.054)
External validation		
Reference model	1.129 (0.613 to 1.598)	1.116 (0.645 to 1.541)
Family-history model	0.895 (0.421 to 1.324)	0.924 (0.492 to 1.317)
At-pregnancy-confirmation model	1.198 (1.090 to 1.309)	1.188 (1.081 to 1.298)
In-early-pregnancy model	1.177 (1.074 to 1.284)	1.156 (1.053 to 1.263)

PRS: polygenic risk score. “No PRS” indicates models without PRS and “M-PRS” indicates models with maternal PRS for diastolic blood pressure. Reference models included maternal age. Family-history models included maternal family history of hypertensive disorders of pregnancy and maternal age. At-pregnancy-confirmation models included variables available at pregnancy confirmation: maternal age, pre-pregnancy body mass index, chronic hypertension, systemic lupus erythematosus, diabetes mellites, maternal family history of hypertensive disorders of pregnancy, conception via in vitro fertilization, parity, gestational age at the previous delivery, and the inter-birth interval. At-early-pregnancy models added blood pressure in early pregnancy to at-pregnancy-confirmation models. Models with M-PRS also included maternal four genetic principal components. Model training and internal validation were conducted using the internal validation cohort. Apparent performance, which reflects the raw performance of the model trained on the internal validation cohort and may be overestimated, was also reported. External validation was conducted using the external validation cohort. *P*-values and 95% confidence intervals were obtained by the bootstrap method with 5,000 resamplings.

**Table 5:** Prediction performance with parental polygenic risk score, baseline characteristics, and blood pressure in early pregnancy in the parental cohorts.

	No PRS	M-PRS	*P*-PRS	M-PRS and *P*-PRS
C-statistics				
Apparent				
Reference model	0.567 (0.529 to 0.605)	0.580 (0.537 to 0.624), *P*-value = 0.435	0.609 (0.567 to 0.651), *P*-value = 0.099	0.608 (0.568 to 0.650), *P*-value = 0.141
Family-history model	0.572 (0.533 to 0.610)	0.583 (0.538 to 0.626), *P*-value = 0.518	0.614 (0.571 to 0.655), *P*-value = 0.198	0.614 (0.572 to 0.655), *P*-value = 0.245
At-pregnancy-confirmation model	0.723 (0.679 to 0.764)	0.728 (0.686 to 0.769), *P*-value = 0.560	0.737 (0.694 to 0.780), *P*-value = 0.217	0.738 (0.694 to 0.780), *P*-value = 0.241
In-early-pregnancy model	0.772 (0.731 to 0.810)	0.770 (0.730 to 0.808), *P*-value = 0.657	0.777 (0.736 to 0.816), *P*-value = 0.400	0.776 (0.736 to 0.815), *P*-value = 0.514
Internal validation				
Reference model	0.567 (0.567 to 0.567)	0.568 (0.547 to 0.582), *P*-value = 0.896	0.598 (0.577 to 0.609), *P*-value = 0.018	0.593 (0.573 to 0.606), *P*-value = 0.056
Family-history model	0.572 (0.563 to 0.573)	0.572 (0.552 to 0.584), *P*-value = 0.995	0.601 (0.580 to 0.613), *P*-value = 0.059	0.597 (0.576 to 0.611), *P*-value = 0.142
At-pregnancy-confirmation model	0.711 (0.683 to 0.725)	0.708 (0.680 to 0.726), *P*-value = 0.777	0.721 (0.696 to 0.735), *P*-value = 0.022	0.718 (0.693 to 0.734), *P*-value = 0.095
In-early-pregnancy model	0.763 (0.741 to 0.771)	0.758 (0.736 to 0.768), *P*-value = 0.160	0.761 (0.738 to 0.773), *P*-value = 0.584	0.759 (0.736 to 0.771), *P*-value = 0.915
External validation				
Reference model	0.610 (0.493 to 0.725)	0.635 (0.495 to 0.753), *P*-value = 0.568	0.504 (0.357 to 0.650), *P*-value = 0.006	0.538 (0.395 to 0.675), *P*-value = 0.055
Family-history model	0.600 (0.484 to 0.713)	0.626 (0.489 to 0.743), *P*-value = 0.549	0.495 (0.349 to 0.641), *P*-value = 0.008	0.530 (0.387 to 0.666), *P*-value = 0.070
At-pregnancy-confirmation model	0.937 (0.858 to 0.985)	0.939 (0.865 to 0.984), *P*-value = 0.410	0.938 (0.868 to 0.981), *P*-value = 0.845	0.936 (0.868 to 0.980), *P*-value = 0.802
In-early-pregnancy model	0.979 (0.963 to 0.989)	0.977 (0.960 to 0.988), *P*-value = 0.031	0.977 (0.961 to 0.987), *P*-value = 0.084	0.973 (0.956 to 0.985), *P*-value = 0.010
Calibration slope				
Apparent				
Reference model	0.995 (0.495 to 1.462)	1.001 (0.574 to 1.402)	0.999 (0.653 to 1.348)	1.000 (0.681 to 1.315)
Family-history model	0.995 (0.515 to 1.448)	0.997 (0.580 to 1.392)	0.999 (0.647 to 1.338)	0.999 (0.680 to 1.313)
At-pregnancy-confirmation model	1.000 (0.850 to 1.164)	1.000 (0.852 to 1.161)	0.998 (0.854 to 1.164)	0.999 (0.853 to 1.161)
In-early-pregnancy model	1.001 (0.864 to 1.151)	1.001 (0.867 to 1.147)	1.001 (0.867 to 1.150)	1.001 (0.869 to 1.146)
Internal validation				
Reference model	1.005 (0.684 to 2.026)	0.805 (0.574 to 1.213)	0.822 (0.613 to 1.156)	0.752 (0.575 to 1.009)
Family-history model	0.959 (0.671 to 1.682)	0.810 (0.596 to 1.190)	0.782 (0.128 to 1.097)	0.729 (0.170 to 0.976)
At-pregnancy-confirmation model	0.918 (0.601 to 1.079)	0.899 (0.588 to 1.054)	0.871 (0.487 to 1.037)	0.856 (0.463 to 1.016)
In-early-pregnancy model	0.921 (0.661 to 1.074)	0.905 (0.651 to 1.048)	0.881 (0.558 to 1.036)	0.866 (0.556 to 1.012)
External validation				
Reference model	1.181 (-0.230 to 2.138)	0.973 (-0.308 to 1.931)	0.284 (-0.953 to 1.261)	0.287 (-0.806 to 1.192)
Family-history model	0.844 (-0.421 to 1.670)	0.715 (-0.416 to 1.543)	0.159 (-1.033 to 1.016)	0.177 (-0.862 to 0.983)
At-pregnancy-confirmation model	1.563 (1.313 to 1.868)	1.525 (1.281 to 1.836)	1.515 (1.269 to 1.835)	1.479 (1.235 to 1.798)
In-early-pregnancy model	1.479 (1.261 to 1.798)	1.412 (1.202 to 1.723)	1.450 (1.226 to 1.780)	1.385 (1.168 to 1.709)

PRS: polygenic risk score. “No PRS,” “M-PRS,” “P-PRS,” and “M-PRS and P-PRS” indicate models without PRS, models with maternal PRS for diastolic blood pressure, models with paternal PRS for diastolic blood pressure, and models with both maternal and paternal PRS for diastolic blood pressure, respectively. Reference models included maternal age. Family-history models included maternal family history of hypertensive disorders of pregnancy and maternal age. At-pregnancy-confirmation models included variables available at pregnancy confirmation: maternal age, pre-pregnancy body mass index, chronic hypertension, systemic lupus erythematosus, diabetes mellitus, maternal family history of hypertensive disorders of pregnancy, conception by in vitro fertilization, parity, gestational age at the previous delivery, and the inter-birth interval. At-early-pregnancy models added blood pressure in early pregnancy to at-pregnancy-confirmation models. Paternal age at conception was included in all the models with paternal PRS and paternal family history of hypertensive disorders of pregnancy was included in all the models with paternal PRS except for reference models. Models with M-PRS and P-PRS also included maternal and paternal four genetic principal components, respectively. Model training and internal validation were conducted using the internal validation cohort. Apparent performance, which reflects the raw performance of the model trained on the internal validation cohort and may be overestimated, was also reported. External validation was conducted using the external validation cohort. *P*-values and 95% confidence intervals were obtained by the bootstrap method with 5,000 resamplings.

## Electronic supplementary material

Below is the link to the electronic supplementary material.

Supplementary Material 1

## Data Availability

The datasets used during the current study available from the corresponding author on reasonable request.
