# Supplementary material for: Preeclampsia prediction with maternal and paternal polygenic risk scores: the TMM BirThree Cohort Study
Source: Sci Rep. 2025 Apr 21;15:13743. doi: 10.1038/s41598-025-97291-x (PMC12012198; doi:10.1038/s41598-025-97291-x)
Supplement: Supplementary file 1 — Supplementary Material 1 [file 41598_2025_97291_MOESM1_ESM.pdf]

Supplementary Materials

Preeclampsia prediction with maternal and paternal polygenic risk scores: the TMM BirThree Cohort Study

Hisashi OHSETO, MD, Mami ISHIKURO, PhD, Taku OBARA, PhD, Akira NARITA, PhD, Ippei TAKAHASHI, MMSc, Genki SHINODA, MS, Aoi NODA, PhD Keiko MURAKAMI, MPH, PhD, Masatsugu ORUI, MD, PhD, Noriyuki IWAMA, MD, PhD, Masahiro KIKUYA, MD, PhD, Hirohito METOKI, MD, PhD, Junichi SUGAWARA, MD, PhD, Gen TAMIYA, PhD, Shinichi KURIYAMA, MD, PhD

Items		Pages
Supplementary Methods		
	Genotyping and quality control	3
	Polygenic risk score	3
	Statistical analysis	4
Supplementary Tables		
	Supplementary Table S1. The number of single-nucleotide polymorphisms included in each polygenic risk score	5
	Supplementary Table S2. Baseline characteristics in the parental internal and external cohort, stratified by the preeclampsia status	6
	Supplementary Table S3. Baseline characteristics in the maternal cohort, stratified by internal and external validation cohorts	7
	Supplementary Table S4. Baseline characteristics in the parental cohort, stratified by internal and external validation cohorts	8
	Supplementary Table S5. Relationship between parental polygenic risk scores and preeclampsia onset in the parental cohorts by logistic regression analysis	9
	Supplementary Table S6. Relationship between maternal polygenic risk scores and preeclampsia onset in the maternal cohorts by logistic regression analysis after excluding the participants with chronic hypertension	11
	Supplementary Table S7. Relationship between parental polygenic risk scores and preeclampsia onset in the parental cohorts by logistic regression analysis after excluding the participants with chronic hypertension	12
	Supplementary Table S8. Relationship between maternal polygenic risk scores and $\log_{10}$ transformed multiple of the median of mean arterial pressure during early pregnancy in the maternal cohorts by linear regression analysis	14
	Supplementary Table S9. Relationship between maternal polygenic risk scores and early- and late-onset preeclampsia in the maternal cohorts by logistic regression analysis	15
	Supplementary Table S10. Changes in the most predictive polygenic risk score because of split ratios and random numbers	17
Supplementary Figure		
	Supplementary Figure S1. Heatmap of correlation coefficients among parental polygenic risk scores	18

Supplementary Methods

Genotyping and quality control

The preimputation quality controls are detailed elsewhere.[22] For imputation, prephasing was performed using SHAPEIT2,[42] along with the "--duohmm" option[43] to increase the accuracy of phasing by considering relatedness between individuals. The phased genotypes were then imputed using IMPUTE4[44] with the cross-imputed panel of 3.5KJPNv2[45] and 1KGP3[46] created using IMPUTE2[47] with the "-merge_ref_panels" option. Variants with minor allele frequencies less than 0.01 or imputation information scores less than 0.4 were removed after imputation.

Polygenic risk score

The polygenic risk score (PRS) for three phenotypes, namely systolic blood pressure (SBP), diastolic blood pressure (DBP), and preeclampsia (PE), was calculated for each participant. Genome-wide association study (GWAS) summary statistics for PRS calculations were obtained from a comprehensive GWAS meta-analysis of the BioBank Japan, UK Biobank, and FinnGen[37] for SBP-PRS and a multi-ancestry preeclampsia/eclampsia GWAS meta-analysis[11] for PE-PRS. PE-PRS from a comprehensive GWAS meta-analysis of the BioBank Japan, UK Biobank, and FinnGen[8] was also constructed for the simulation study (details provided in the main manuscript). For optimization, PRSs were calculated using two methods, namely genome-wide clumping and thresholding methods (C+T) and a Bayesian approach using the LDpred2 non-sparse grid model (LDpred2), which accounts for linkage disequilibrium between single nucleotide polymorphisms. These two methods have been described elsewhere.[28,29] We restricted the variants to HapMap3 following the

LDpred2 implementation method.[29] Standard quality control was applied to both summary statistics and genotyping data. In the C+T method, the clumping r^2, P-value thresholds, and base clumping window size were optimized in the PRS training cohort. In the LDpred2 method, hyperparameter SNP heritability and the proportion of causal variants in the LDpred2 model were optimized in the PRS training cohort. This optimization was performed based on Harrell's C-statistics using five-fold cross-validation in the PRS training cohort. The PRSs were standardized to a mean of 0 and a standard deviation of 1 and were included in the analysis as continuous variables. Due to the relatively small sample size of paternal participants, we applied the optimized parameters for maternal PRSs without additional re-optimization for paternal PRSs. This approach balances computational feasibility and ensures robustness in PRS calculation.

Statistical analysis

In the maternal internal and external validation cohorts, models with or without maternal PRS and four genetic principal components (PC) were compared. In the internal and external parental validation cohorts, models without PRS, with maternal PRS and four genetic PCs, with paternal PRS and four genetic PCs, and with both maternal and paternal PRSs and four genetic PCs were compared. Paternal age at conception was included in all models with paternal PRS, and paternal family history of hypertensive disorders of pregnancy was included in all models with paternal PRS except for the reference model.

Supplementary Table S1. The number of single-nucleotide polymorphisms included in each polygenic risk score

	LDpred2	C+T
Internal validation cohort		
Systolic blood pressure	801,846	213,644
Diastolic blood pressure	801,846	324,660
Preeclampsia	410,937	50,123
External validation cohort		
Systolic blood pressure	764,750	215,825
Diastolic blood pressure	764,750	325,034
Preeclampsia	392,255	50,935

PRS: polygenic risk score.

Each cell represents the number of SNPs in each PRS, calculated for three phenotypes using two methods, LDpred2 and C+T.

Supplementary Table S2. Baseline characteristics in the parental internal and external cohort, stratified by the preeclampsia status

	Parental internal validation cohort (n = 3,673)				Parental external validation cohort (n = 2,616)			
	PE<br>n = 216	Not affected<br>n = 3,457	P-value	n of missing	PE<br>n = 29	Not affected<br>n = 2,587	P-value	n of missing
Gestational age, weeks	38.6 ± 2.2	39.2 ± 1.6	<0.001	0	38.7 ± 1.9	39.2 ± 1.6	0.085	0
Maternal age at conception, years	32.4 ± 5.5	31.6 ± 4.9	0.024	0	32.8 ± 5.3	31.6 ± 5.0	0.213	0
Pre-pregnancy BMI, kg/m^2	23.5 ± 4.7	21.7 ± 3.5	<0.001	60	23.9 ± 6.1	21.5 ± 3.3	<0.001	33
CH, %	54 (25.0)	88 (2.5)	<0.001	0	23 (79.3)	45 (1.7)	<0.001	0
DM (type 1 or 2), %	5 (2.3)	12 (0.3)	0.002	1,007	0 (0.0)	2 (0.1)	>0.999	866
SLE, %	2 (0.9)	5 (0.1)	0.059	1,007	1 (3.4)	3 (0.1)	0.044	866
Maternal family history of HDP, %	10 (4.6)	85 (2.5)	0.084	1,007	0 (0.0)	46 (1.8)	0.989	866
Parity			<0.001	4			0.026	22
Nulliparous, %	108 (50.0)	1,619 (46.8)			12 (41.4)	1,124 (43.4)		
Parous with previous PE, %	20 (9.3)	85 (2.5)			3 (10.3)	63 (2.4)		
Parous with no previous PE, %	88 (40.7)	1,753 (50.7)			14 (48.3)	1,400 (54.1)		
Inter-birth interval, years	4.0 ± 3.5	3.6 ± 2.7	0.114	74	3.1 ± 1.6	3.8 ± 2.9	0.280	75
Last delivery gestational age, weeks	38.6 ± 2.2	39.0 ± 1.7	0.031	698	38.2 ± 1.8	39.0 ± 1.7	0.060	533
Conception by IVF, %	22 (10.2)	189 (5.5)	0.006	11	2 (6.9)	115 (4.4)	0.855	44
MAP at 10–13 weeks of gestation, mmHg	88.8 ± 10.7	80.5 ± 9.2	<0.001	470	96.5 ± 10.8	79.5 ± 9.2	<0.001	355
Paternal age at conception, years	34.1 ± 6.5	33.2 ± 5.8	0.031	0	34.2 ± 5.7	33.4 ± 5.8	0.449	0
Paternal family history of HDP, %	2 (0.9)	22 (0.6)	0.649	269	0 (0.0)	15 (0.6)	>0.999	246

PE: preeclampsia; BMI: body mass index; CH: chronic hypertension; DM: diabetes mellitus; SLE: systemic lupus erythematosus; HDP: hypertensive disorders of pregnancy; IVF: in vitro fertilization; MAP: mean arterial pressure.

Data are shown as mean ± standard deviation for continuous variables and n (%) for categorical variables.

P-values were calculated using the chi-square or Fisher's exact test for categorical variables and the *t*-test for continuous variables.

Missing values were imputed using k-nearest neighbor imputation with k = 140 (square root of the total study population).

Supplementary Table S3. Baseline characteristics in the maternal cohort, stratified by internal and external validation cohorts

	Internal validation *n* = 6,768	External validation *n* = 9,684	*P*-value
PE, %	352 (5.2)	268 (2.8)	<0.001
Gestational age, weeks	39.2 ± 1.6	39.1 ± 1.8	0.012
Maternal age at conception, years	31.5 ± 5.0	31.4 ± 5.0	0.048
Pre-pregnancy BMI, kg/m^2	21.7 ± 3.5	21.5 ± 3.4	<0.001
CH, %	281 (4.2)	295 (3.0)	<0.001
DM (type 1 or 2), %	25 (0.4)	9 (0.1)	<0.001
SLE, %	7 (0.1)	5 (0.1)	0.251
Maternal family history of HDP, %	148 (2.2)	161 (1.7)	0.017
Parity			<0.001
Nulliparous, %	2,972 (43.9)	3,717 (38.4)	
Parous with previous PE, %	204 (3.0)	289 (3.0)	
Parous with no previous PE, %	3,592 (53.1)	5,678 (58.6)	
Inter-birth interval, years	3.7 ± 2.7	3.6 ± 2.6	0.741
Last delivery gestational age, weeks	39.0 ± 1.6	39.0 ± 1.6	0.587
Conception via IVF, %	331 (4.9)	434 (4.5)	0.235
MAP at 10–13 weeks of gestation, mmHg	80.9 ± 9.6	80.0 ± 9.6	<0.001

PE: preeclampsia; BMI: body mass index; CH: chronic hypertension; DM: diabetes mellitus; SLE: systemic lupus erythematosus; HDP: hypertensive disorders of pregnancy; IVF: in vitro fertilization; MAP: mean arterial pressure.

Data are shown as mean ± standard deviation for continuous variables and n (%) for categorical variables. *P*-values were calculated using the chi-square or Fisher's exact test for categorical variables and the *t*-test for continuous variables.

Supplementary Table S4. Baseline characteristics in the parental cohort, stratified by internal and external validation cohorts

	Internal validation n = 3,673	External validation n = 2,616	P-value
PE, %	216 (5.9)	29 (1.1)	<0.001
Gestational age, weeks	39.2 ± 1.6	39.2 ± 1.6	0.940
Maternal age at conception, years	31.6 ± 4.9	31.6 ± 5.0	0.928
Pre-pregnancy BMI, kg/m^2	21.8 ± 3.6	21.5 ± 3.4	<0.001
CH, %	142 (3.9)	68 (2.6)	0.007
DM (type 1 or 2), %	17 (0.5)	2 (0.1)	0.005
SLE, %	7 (0.2)	4 (0.2)	>0.999
Maternal family history of HDP, %	95 (2.6)	46 (1.8)	0.036
Parity			0.009
Nulliparous, %	1,727 (47.0)	1,136 (43.4)	
Parous with previous PE, %	105 (2.9)	66 (2.5)	
Parous with no previous PE, %	1,841 (50.1)	1,414 (54.1)	
Inter-birth interval, years	3.6 ± 2.7	3.8 ± 2.9	0.039
Last delivery gestational age, weeks	39.0 ± 1.6	39.0 ± 1.6	0.798
Conception via IVF, %	211 (5.7)	117 (4.5)	0.029
MAP at 10–13 weeks of gestation, mmHg	81.0 ± 9.5	79.6 ± 9.4	<0.001
Paternal age at conception, years	33.3 ± 5.8	33.4 ± 5.8	0.648
Paternal family history of HDP, %	24 (0.7)	15 (0.6)	0.747

PE: preeclampsia; BMI: body mass index; CH: chronic hypertension; DM: diabetes mellitus; SLE: systemic lupus erythematosus; HDP: hypertensive disorders of pregnancy; IVF: in vitro fertilization; MAP: mean arterial pressure.

Data are shown as mean ± standard deviation for continuous variables and *n* (%) for categorical variables. *P*-values were calculated using the chi-square or Fisher's exact test for categorical variables and the *t*-test for continuous variables.

Supplementary Table S5. Relationship between parental polygenic risk scores and preeclampsia onset in the parental cohorts by logistic regression analysis

	Internal validation cohort		External validation cohort		Meta-analysis		
	OR (95% CI)	*P*-value	OR (95% CI)	*P*-value	OR (95% CI)	*P*-value	P for heterogeneity
**Maternal**							
PRS for SBP							
Continuous	0.98 (0.85 to 1.13)	0.763	1.13 (0.77 to 1.66)	0.520	1.00 (0.87 to 1.14)	0.951	0.479
Tertile 1	Reference		Reference		Reference		
Tertile 2	0.94 (0.67 to 1.34)	0.743	0.87 (0.34 to 2.18)	0.761	0.93 (0.67 to 1.29)	0.679	0.866
Tertile 3	0.76 (0.54 to 1.08)	0.126	1.40 (0.58 to 3.34)	0.452	0.83 (0.60 to 1.14)	0.254	0.206
PRS for DBP							
Continuous	1.07 (0.93 to 1.24)	0.341	1.41 (0.94 to 2.11)	0.093	1.11 (0.97 to 1.27)	0.144	0.207
Tertile 1	Reference		Reference		Reference		
Tertile 2	0.73 (0.50 to 1.08)	0.113	1.47 (0.59 to 3.66)	0.404	0.81 (0.57 to 1.16)	0.258	0.165
Tertile 3	0.99 (0.70 to 1.39)	0.952	2.40 (0.96 to 5.99)	0.061	1.10 (0.80 to 1.52)	0.548	0.076
PRS for PE							
Continuous	1.01 (0.88 to 1.16)	0.878	1.36 (0.93 to 1.99)	0.111	1.05 (0.92 to 1.19)	0.488	0.150
Tertile 1	Reference		Reference		Reference		
Tertile 2	1.04 (0.75 to 1.44)	0.814	1.72 (0.59 to 5.00)	0.316	1.09 (0.79 to 1.49)	0.603	0.374
Tertile 3	1.03 (0.73 to 1.46)	0.853	1.82 (0.64 to 5.13)	0.261	1.09 (0.79 to 1.51)	0.596	0.313
**Paternal**							
PRS for SBP							
Continuous	0.94 (0.82 to 1.09)	0.416	1.93 (1.32 to 2.83)	<0.001	1.03 (0.90 to 1.17)	0.693	0.001
Tertile 1	Reference		Reference		Reference		
Tertile 2	0.93 (0.66 to 1.32)	0.691	3.33 (1.05 to 10.53)	0.040	1.04 (0.74 to 1.45)	0.827	0.038
Tertile 3	0.91 (0.64 to 1.27)	0.567	5.94 (1.94 to 18.20)	0.002	1.06 (0.77 to 1.47)	0.717	0.002
PRS for DBP							
Continuous	1.00 (0.87 to 1.16)	0.975	1.91 (1.29 to 2.84)	0.001	1.08 (0.94 to 1.24)	0.260	0.003
Tertile 1	Reference		Reference		Reference		
Tertile 2	1.03 (0.71 to 1.49)	0.890	1.84 (0.68 to 4.99)	0.230	1.10 (0.78 to 1.56)	0.583	0.282
Tertile 3	0.98 (0.69 to 1.40)	0.923	4.89 (1.93 to 12.41)	<0.001	1.21 (0.86 to 1.68)	0.270	0.002
PRS for PE							
Continuous	0.90 (0.78 to 1.04)	0.157	1.09 (0.75 to 1.58)	0.646	0.92 (0.81 to 1.06)	0.246	0.350

Tertile 1	Reference		Reference		Reference		
Tertile 2	0.90 (0.65 to 1.24)	0.501	1.83 (0.69 to 4.85)	0.226	0.96 (0.71 to 1.31)	0.796	0.174
Tertile 3	0.78 (0.55 to 1.11)	0.165	1.25 (0.45 to 3.46)	0.672	0.82 (0.59 to 1.14)	0.240	0.395

PRS: polygenic risk score; PE: preeclampsia; OR: odds ratio; CI: confidence interval; SBP: systolic blood pressure; DBP: diastolic blood pressure.

Two models were developed, one with PRS as a continuous value and the other as tertile values.

All results were adjusted for parental age at conception and four genetic principal components.

P for heterogeneity was calculated by Cochran Q test.

Supplementary Table S6. Relationship between maternal polygenic risk scores and preeclampsia onset in the maternal cohorts by logistic regression analysis after excluding the participants with chronic hypertension

	Internal validation cohort		External validation cohort		Meta-analysis		
	OR (95% CI)	*P*-value	OR (95% CI)	*P*-value	OR (95% CI)	*P*-value	P for heterogeneity
PRS for SBP							
Continuous	0.99 (0.87 to 1.12)	0.871	1.33 (1.13 to 1.55)	<0.001	1.11 (1.01 to 1.23)	0.039	0.005
Tertile 1	Reference		Reference		Reference		
Tertile 2	1.13 (0.82 to 1.56)	0.463	1.06 (0.73 to 1.56)	0.753	1.10 (0.86 to 1.41)	0.445	0.816
Tertile 3	0.89 (0.64 to 1.23)	0.465	1.62 (1.12 to 2.35)	0.011	1.15 (0.90 to 1.47)	0.256	0.016
PRS for DBP							
Continuous	1.08 (0.94 to 1.23)	0.270	1.41 (1.20 to 1.66)	<0.001	1.20 (1.08 to 1.33)	<0.001	0.012
Tertile 1	Reference		Reference		Reference		
Tertile 2	0.78 (0.55 to 1.11)	0.173	1.29 (0.88 to 1.88)	0.190	0.99 (0.76 to 1.28)	0.917	0.059
Tertile 3	1.00 (0.73 to 1.37)	0.998	2.08 (1.43 to 3.03)	<0.001	1.35 (1.06 to 1.72)	0.014	0.004
PRS for PE							
Continuous	1.03 (0.90 to 1.17)	0.666	1.16 (0.99 to 1.36)	0.063	1.08 (0.98 to 1.19)	0.131	0.246
Tertile 1	Reference		Reference		Reference		
Tertile 2	1.03 (0.76 to 1.39)	0.843	1.40 (0.92 to 2.14)	0.116	1.14 (0.90 to 1.46)	0.282	0.244
Tertile 3	1.04 (0.76 to 1.43)	0.798	1.46 (0.97 to 2.20)	0.068	1.18 (0.92 to 1.52)	0.188	0.198

PRS: polygenic risk score; PE: preeclampsia; OR: odds ratio; CI: confidence interval; SBP: systolic blood pressure; DBP: diastolic blood pressure.
Two models were developed, one with PRS as a continuous value and the other as tertile values.
All results were adjusted for maternal age at conception and four genetic principal components.
P for heterogeneity was calculated by Cochran Q test.

Supplementary Table S7. Relationship between parental polygenic risk scores and preeclampsia onset in the parental cohorts by logistic regression analysis after excluding the participants with chronic hypertension

	Internal validation cohort		External validation cohort		Meta-analysis		
	OR (95% CI)	*P*-value	OR (95% CI)	*P*-value	OR (95% CI)	*P*-value	P for heterogeneity
**Maternal**							
PRS for SBP							
Continuous	0.93 (0.79 to 1.09)	0.363	0.73 (0.32 to 1.67)	0.456	0.92 (0.78 to 1.08)	0.301	0.576
Tertile 1	Reference		-		-		
Tertile 2	1.03 (0.70 to 1.51)	0.900	-		-		
Tertile 3	0.65 (0.43 to 0.98)	0.039	-		-		
PRS for DBP							
Continuous	1.04 (0.88 to 1.23)	0.651	1.12 (0.47 to 2.67)	0.801	1.04 (0.89 to 1.22)	0.623	0.870
Tertile 1	Reference		Reference		Reference		
Tertile 2	0.77 (0.50 to 1.18)	0.228	1.03 (0.17 to 6.47)	0.971	0.78 (0.51 to 1.19)	0.244	0.754
Tertile 3	0.91 (0.61 to 1.35)	0.640	0.98 (0.10 to 10.00)	0.989	0.91 (0.62 to 1.34)	0.643	0.948
PRS for PE							
Continuous	1.05 (0.89 to 1.23)	0.578	1.94 (0.81 to 4.64)	0.135	1.07 (0.91 to 1.25)	0.414	0.170
Tertile 1	Reference		Reference		Reference		
Tertile 2	1.13 (0.77 to 1.65)	0.530	0.83 (0.05 to 13.39)	0.894	1.12 (0.77 to 1.63)	0.545	0.828
Tertile 3	1.12 (0.76 to 1.67)	0.563	2.87 (0.31 to 26.18)	0.351	1.16 (0.78 to 1.71)	0.463	0.414
**Paternal**							
PRS for SBP							
Continuous	0.92 (0.79 to 1.08)	0.331	2.74 (1.13 to 6.60)	0.025	0.96 (0.82 to 1.12)	0.580	0.017
Tertile 1	Reference		-		-		
Tertile 2	1.03 (0.69 to 1.54)	0.873	-		-		
Tertile 3	0.87 (0.59 to 1.30)	0.505	-		-		

PRS for DBP							
Continuous	1.02 (0.87 to 1.21)	0.805	2.49 (1.07 to 5.77)	0.034	1.06 (0.90 to 1.24)	0.514	0.042
Tertile 1	Reference		Reference		Reference		
Tertile 2	1.17 (0.76 to 1.81)	0.471	1.35 (0.08 to 22.15)	0.832	1.18 (0.77 to 1.80)	0.456	0.921
Tertile 3	1.09 (0.72 to 1.65)	0.693	9.76 (1.06 to 89.77)	0.044	1.17 (0.78 to 1.77)	0.447	0.057
PRS for PE							
Continuous	0.85 (0.73 to 1.00)	0.054	1.11 (0.47 to 2.62)	0.806	0.86 (0.73 to 1.01)	0.065	0.548
Tertile 1	Reference		Reference		Reference		
Tertile 2	0.80 (0.55 to 1.16)	0.231	0.83 (0.12 to 6.02)	0.856	0.80 (0.56 to 1.15)	0.226	0.967
Tertile 3	0.68 (0.46 to 1.02)	0.063	0.80 (0.11 to 5.88)	0.830	0.69 (0.46 to 1.02)	0.062	0.874

PRS: polygenic risk score; PE: preeclampsia; OR: odds ratio; CI: confidence interval; SBP: systolic blood pressure; DBP: diastolic blood pressure.

Two models were developed, one with PRS as a continuous value and the other as tertile values.

All results were adjusted for parental age at conception and four genetic principal components.

"-" indicates results that did not converge.

P for heterogeneity was calculated by Cochran Q test.

Supplementary Table S8. Relationship between maternal polygenic risk scores and $\log_{10}$ transformed multiple of the median of mean arterial pressure during early pregnancy in the maternal cohorts by linear regression analysis

	Internal validation cohort		External validation cohort	
	Beta (95% CI)	*P*-value	Beta (95% CI)	*P*-value
PRS for SBP				
Continuous	0.08 (-0.03 to 0.19)	0.164	0.32 (0.2 to 0.45)	<0.001
Tertile 1	Reference		Reference	
Tertile 2	0.08 (-0.18 to 0.33)	0.558	0.78 (0.48 to 1.08)	<0.001
Tertile 3	-0.27 (-0.56 to 0.02)	0.066	0.33 (0.02 to 0.64)	0.038
PRS for DBP				
Continuous	0.02 (-0.08 to 0.13)	0.647	0.27 (0.15 to 0.4)	<0.001
Tertile 1	Reference		Reference	
Tertile 2	-0.04 (-0.3 to 0.23)	0.785	0.41 (0.11 to 0.7)	0.007
Tertile 3	0.02 (-0.25 to 0.29)	0.879	0.05 (-0.26 to 0.36)	0.740
PRS for PE				
Continuous	0.02 (-0.09 to 0.13)	0.765	0.19 (0.07 to 0.31)	0.002
Tertile 1	Reference		Reference	
Tertile 2	0.06 (-0.2 to 0.33)	0.635	0.45 (0.12 to 0.77)	0.008
Tertile 3	0.07 (-0.19 to 0.32)	0.599	0.43 (0.10 to 0.77)	0.012

PRS: polygenic risk score; CI: confidence interval; SBP: systolic blood pressure; DBP: diastolic blood pressure.
Two models were developed, one with PRS as a continuous value and the other as tertile values.
All results were adjusted for maternal age at conception and four genetic principal components.

Supplementary Table S9. Relationship between maternal polygenic risk scores and early- and late-onset preeclampsia in the maternal cohorts by logistic regression analysis

	Internal validation cohort		External validation cohort	
	OR (95% CI)	*P*-value	OR (95% CI)	*P*-value
Early onset PE				
PRS for SBP				
Continuous	1.06 (0.88 to 3.05)	0.521	1.41 (1.17 to 1.1)	<0.001
Tertile 1	Reference		Reference	
Tertile 2	0.94 (0.58 to 6.35)	0.816	1.23 (0.76 to 2.84)	0.407
Tertile 3	1.1 (0.7 to 4.67)	0.668	2.42 (1.56 to 1.25)	<0.001
PRS for DBP				
Continuous	1.03 (0.86 to 5.07)	0.781	1.36 (1.13 to 1.1)	0.001
Tertile 1	Reference		Reference	
Tertile 2	1.07 (0.68 to 5.79)	0.778	1.06 (0.66 to 6.21)	0.81
Tertile 3	0.94 (0.6 to 6.11)	0.805	1.52 (0.98 to 1.41)	0.062
PRS for PE				
Continuous	0.98 (0.82 to 5.91)	0.858	1.16 (0.96 to 1.38)	0.116
Tertile 1	Reference		Reference	
Tertile 2	1.36 (0.89 to 1.7)	0.16	1.15 (0.7 to 3.96)	0.574
Tertile 3	0.96 (0.59 to 6.87)	0.856	1.35 (0.85 to 1.9)	0.207
Late onset PE				
PRS for SBP				
Continuous	1.09 (0.96 to 1.59)	0.201	1.36 (1.15 to 1.09)	<0.001
Tertile 1	Reference		Reference	
Tertile 2	0.68 (0.48 to 1.28)	0.033	1.53 (1.02 to 1.33)	0.042
Tertile 3	1.07 (0.79 to 4.33)	0.668	2 (1.33 to 1.23)	0.001

PRS for DBP					
	Continuous	1.03 (0.9 to 4.26)	0.706	1.28 (1.09 to 1.09)	0.003
	Tertile 1	Reference		Reference	
	Tertile 2	1 (0.72 to 8.24)	0.992	1.05 (0.69 to 6.07)	0.813
	Tertile 3	0.97 (0.71 to 6.46)	0.869	1.5 (1.01 to 1.33)	0.044
PRS for PE					
	Continuous	1.03 (0.91 to 3.62)	0.622	1.25 (1.06 to 1.1)	0.007
	Tertile 1	Reference		Reference	
	Tertile 2	0.95 (0.69 to 4.87)	0.726	1.96 (1.23 to 1.28)	0.004
	Tertile 3	1.12 (0.81 to 3.08)	0.492	1.8 (1.13 to 1.3)	0.012

PRS: polygenic risk score; PE: preeclampsia; OR: odds ratio; CI: confidence interval; SBP: systolic blood pressure; DBP: diastolic blood pressure.

Two models were developed, one with PRS as a continuous value and the other as tertile values.

All results were adjusted for maternal age at conception and four genetic principal components.

Supplementary Table S10. Changes in the most predictive polygenic risk score because of split ratios and random numbers

	Split ratio	Systolic blood pressure	Diastolic blood pressure	Preeclampsia*	Preeclampsia†
Internal validation	1:2	52	31	6	11
	2:3	46	32	7	14
	1:1	41	35	10	14
External validation	1:2	15	81	3	1
	2:3	17	77	3	2
	1:1	20	68	9	3

*The PRS was generated from the GWAS summary statistics cited in number 9 in Supplementary Reference.

†The PRS was generated from the GWAS summary statistics cited in number 8 in Supplementary Reference.

For each ratio, the JPA v2 cohort was divided into the PRS train cohort and the internal validation cohort. The trials were repeated with 100 random numbers. Cells indicate the number of times the PRS had the best predictive power in each internal and external cohort; for Preeclampsia, two PRSs were generated from two GWAS summaries.

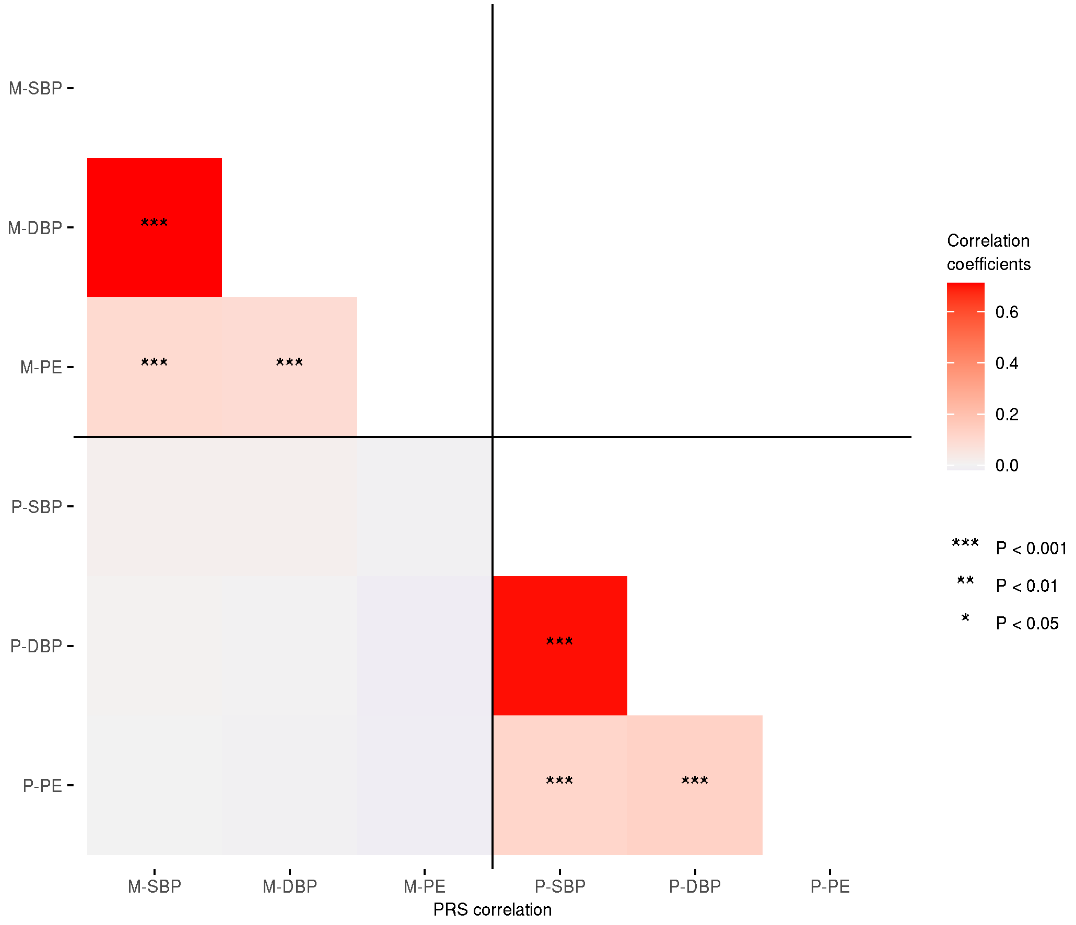

Supplementary Figure S1. Heatmap of correlation coefficients among parental polygenic risk scores

Parental PRSs were calculated for three phenotypes, PE, SBP, and DBP, using LDpred2. Pearson's correlation coefficients are indicated by colors, where red indicates a positive correlation, blue indicates a negative correlation, and gray indicates no correlation. The asterisks indicate the *P*-values for the correlation coefficients.

PRS: polygenic risk score; M: maternal; P: paternal; PE: preeclampsia; SBP: systolic blood pressure; DBP: diastolic blood pressure.
